# Sex differences in the presentation and management of acute coronary syndrome patients: Insights from the FORCE-ACS registry

**DOI:** 10.1016/j.ijcha.2025.101849

**Published:** 2025-12-10

**Authors:** Shabiga Sivanesan, Aleksandra Gąsecka, Niels M.R. van der Sangen, Wout W.A. van den Broek, Jaouad Azzahhafi, Dean R.P.P. Chan Pin Yin, Qiu Ying F. van de Pol, Ronald J. Walhout, Melvyn Tjon Joe Gin, Ron Pisters, Deborah M. Nicastia, Gerben J. de Roest, Georgios J. Vlachojannis, Rutger J. van Bommel, Wouter J. Kikkert, José P.S. Henriques, Jurriën M. ten Berg, Yolande Appelman

**Affiliations:** a Department of Cardiology, Amsterdam UMC, University of Amsterdam, Amsterdam Cardiovascular Sciences, Amsterdam, the Netherlands; b Department of Cardiology, Amsterdam UMC, VU University, Amsterdam Cardiovascular Sciences, Amsterdam, the Netherlands; c Department of Cardiology, St. Antonius Hospital, Nieuwegein, the Netherlands; d Department of Cardiology, Hospital Gelderse Vallei, Ede, the Netherlands; e Department of Cardiology, Rijnstate Hospital, Arnhem, the Netherlands; f Department of Cardiology, Gelre Hospitals, Apeldoorn, the Netherlands; g Department of Cardiology, Rivierenland Hospital, Tiel, the Netherlands; h Department of Cardiology, University Medical Center Utrecht, Utrecht, the Netherlands; i Department of Cardiology, Tergooi MC, Hilversum, the Netherlands; j Department of Cardiology, University Medical Center Maastricht, Maastricht, the Netherlands

**Keywords:** Acute coronary syndrome, Men, Sex differences, Registry, Women

## Abstract

**Aims:**

This study reports sex differences in the clinical presentation, treatment management and outcomes of patients with acute coronary syndrome (ACS) in The Netherlands, using data from the FORCE-ACS registry.

**Methods:**

A prospective analysis was conducted using data from 5023 patients admitted with ACS between 2015 and 2019, with complete three-year follow-up. Demographic data, clinical characteristics, in-hospital treatment and outcomes were compared by sex. Multivariable regression analyses explored associations between sex and clinical outcomes.

**Results:**

Of the 5023 patients, 29 % were women. Women were generally older, with a significantly higher prevalence of hypertension (61.7 % vs 54.2 %), chronic kidney disease (25.7 % vs. 18.5 %) and myocardial infarction with non-obstructive coronary arteries (MINOCA) (13.5 % vs. 6.5 %). Women less frequently underwent revascularisation, even after excluding those with non-obstructive coronary artery disease, and received less medical treatment compared to their male counterparts. At 36 months, women had higher unadjusted mortality rate (13.7 % vs. 11.0 %, OR 1.28, 95 % CI: 1.07–1.54) and bleeding events (26.2 % vs. 22.3 %, OR 1.24, 95 % CI: 1.08–1.43). However, after adjustment for age and baseline characteristics, these differences were no longer statistically significant. Recurrent ACS and stroke remained similar in both groups, also after correction.

**Conclusion:**

Differences between women and men were observed in clinical presentation, interventional treatment, pharmacotherapy and outcomes among ACS patients in The Netherlands. Despite receiving less guideline-recommended care, women had similar adjusted 36-month outcomes as men. These findings show that there is room for improvement in the management of ACS, with a focus on optimized treatment strategies for women.

## Graphical abstract

**Figure:**
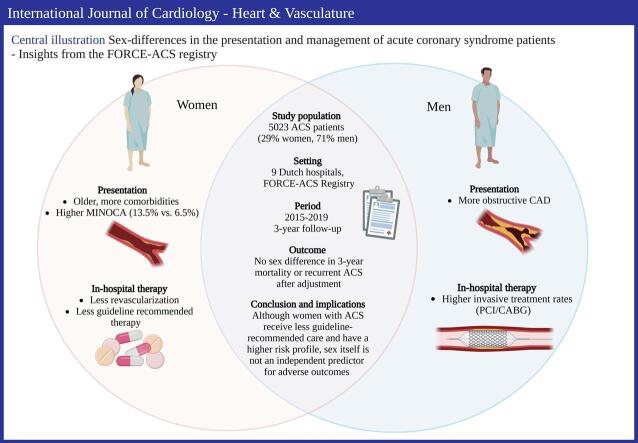

## 1 Introduction

Acute coronary syndrome (ACS) accounts for 1.8 million deaths each year, making it one of the leading causes of mortality for both women and men worldwide [1]. Despite significant advancements in its diagnosis and treatment, more than one in five patients experience a recurrent ischemic event within five years [1]. One of the underlying reasons is inadequate secondary prevention, including insufficient implementation of guideline-recommended care [2]. Studies have demonstrated that women with ACS are less likely to be treated with guideline-recommended care compared to their male counterparts [3,4]. A recent study conducted in The Netherlands showed that women were about 25 % more likely to be classified as high risk, while they were 29 % less likely to receive optimal guideline-recommended care compared to men, even after adjustments for demographic and clinical characteristics [5]. Although national and international guidelines clearly define (the state-of-the art) diagnostic and therapeutic algorithms for ACS patients, these guidelines do not specifically address sex differences [2]. Thus, to address the knowledge gap in sex differences in the presentation, management and outcomes of patients with ACS, we conducted a comparative analysis of clinical characteristics, treatment strategies and outcomes in patients enrolled in the Future Optimal Research and Care Evaluation in Patients with Acute Coronary Syndrome (FORCE-ACS) registry.

## 2 Methods

### 2.1 Study design and patient population

The rationale and design of the FORCE-ACS registry have been described previously [6]. In brief, the FORCE-ACS registry is an ongoing prospective registry of nine Dutch hospitals. The primary aim of the registry is to provide insight into different aspects of the diagnosis, management and follow-up of ACS patients. Patient management, including the use of invasive and pharmacological therapies, in all participating hospitals is performed according to relevant guidelines during that period [7,8]. From 2015 onwards, all consecutive adult patients admitted for (suspected) ACS (i.e., ST-segment elevation myocardial infarction (STEMI), non-ST-segment elevation myocardial infarction (NSTEMI) or unstable angina) were eligible for participation. For the present study, all patients who were diagnosed with ACS during index hospital admission from January 2015 until December 2019 and who completed three years follow-up, were included. The institutional review boards of all participating centres approved the protocol of the FORCE-ACS registry and written consent was obtained from each patient. The current study complies with the principles of the Declaration of Helsinki and reports according to the STrengthening the Reporting of OBservational studies in Epidemiology (STROBE) statement [9].

### 2.2 Clinical presentation and patient management

Clinical characteristics and data regarding patient management were extracted from the electronic health records (EHRs) manually or using an automated software to minimize errors due to manual data extraction, if allowed by the participating hospital. Optimal care was defined as administration of all five Class I guideline-recommended medications (i.e. acetylsalicylic acid (ASA), P2Y_12_-inhibitor, β-blocker, angiotensin-converting enzyme (ACE) inhibitor or angiotensin receptor blocker (ARB) and cholesterol-lowering drug). Regarding antithrombotic therapy, dual antiplatelet therapy (DAPT) was defined as a combination of ASA and a P2Y_12_-inhibitor, whereas single antiplatelet therapy (SAPT) was defined as the use of either ASA or P2Y_12_-inhibitor alone. In patients with an indication for an oral anticoagulation (OAC), ASA and/or a P2Y_12_-inhibitor on top of OAC was also considered as optimal care, defined as dual antithrombotic therapy (DAT) or triple antithrombotic therapy (TAT) [2]. DAPT, DAT and TAT were all considered as optimal antithrombotic therapy.

### 2.3 Follow-up

Clinical events were reported via questionnaires at 1, 12, 24 and 36 month(s) after initial hospital admission. If patients did not complete the questionnaire, they were contacted by phone. Additionally, the EHRs of all patients were checked for possible events. In case of clinical event, relevant source document was collected. Clinical events were reviewed and adjudicated by the coordinating investigators of the FORCE-ACS Registry.

### 2.4 Endpoint and definitions

The primary endpoint was all-cause death at 36 months. Secondary endpoints included cardiovascular death, non-cardiovascular death, recurrent ACS, stroke, major bleeding and/or minor bleeding at 36 months. Death was defined according to the recent Academic Research Consortium, ACS and Myocardial Infarction with Non-Obstructive Coronary Arteries (MINOCA) was defined according to The Fourth Universal Definition of Myocardial Infarction [10,11]. MINOCA was further defined as MI with < 50 % stenosis in all major epicardial coronary arteries. Major bleeding was classified as Bleeding Academic Research Consortium (BARC) type 3 or 5, whereas minor bleeding was classified as BARC type 2.

### 2.5 Statistical analysis methods

Continuous variables were reported as mean ± standard deviation or median and interquartile range (IQR), whereas categorical variables were reported as frequencies and percentages. Patient characteristics were compared per sex using an independent *t*-test for continuous variables and chi-square test for categorical variables. Sex differences in clinical outcomes (e.g. death, recurrent ACS, stroke and bleeding) were evaluated using univariable and age-adjusted logistic regression models to explore associations. This was followed by multivariable models adjusting for baseline characteristics that significantly differed between women and men. Significance was set at a p-value of < 0.05. Statistical analyses were performed using SPSS version 28 (SPSS Inc., Chicago, IL) and illustrative graphics were composed using Biorender.

## 3 Results

**Fig. 1:**
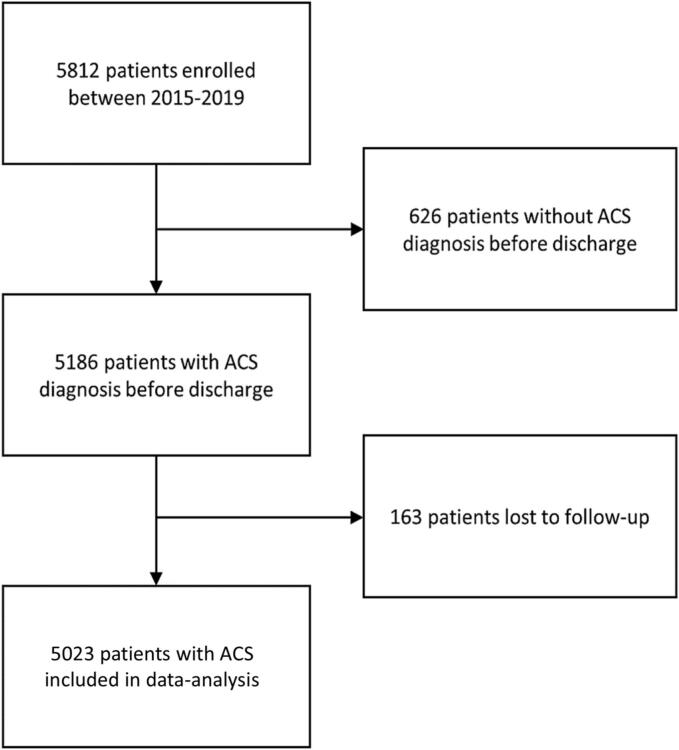
Flowchart **ACS** denotes acute coronary syndrome.

From January 2015 until December 2019, 5812 patients (1753 women and 4059 men) were included in the FORCE-ACS registry. Patients who were ultimately not diagnosed with ACS at discharge (n = 626) or who did not complete three-years follow-up (n = 163) were excluded. In total, 5023 patients were included in the present analysis (1463 women (29 %) and 3560 men (71 %)). A flowchart is provided in Fig. 1.

### 3.1 Clinical characteristics

The clinical characteristics of women and men during hospital admission are shown in Table 1. Women were generally older than men (mean age 69.7 ± 12.4 years vs. 65.5 ± 11.7 years, P < 0.001) and more frequently had hypertension (61.7 % vs. 54.2 %, P < 0.001) or chronic kidney disease, defined as a glomerular filtration rate less than 60 ml/min/1.73 m^2^ (25.7 % vs. 18.5 %, P < 0.001). However, women were less likely to be smokers (49.8 % vs. 62.3 %, P < 0.001), had a lower incidence of previous myocardial infarction (MI) (18.0 % vs. 23.9 %, P < 0.001) and were less likely to have undergone previous percutaneous coronary intervention (PCI) (18.1 % vs. 23.5 %, P < 0.001). Women more often had comorbidities: atrial fibrillation, diabetes, peripheral artery disease, chronic kidney disease or chronic obstructive pulmonary disease (COPD) (49.0 % vs. 42.6 % P < 0.001). Regardless of sex, most patients presented with NSTEMI (52.8 % of women vs. 49.9 % of men), followed by STEMI (37.9 % vs. 41.4 %) and unstable angina (9.2 % vs. 8.7 %). Women were more likely to present with non-obstructive coronary artery disease (13.5 % vs. 6.5 %, P < 0.001) and had a higher prevalence of Killip class ≥ II at admission compared to men (15.3 % vs. 11.2 %, P < 0.001). Most patients had a left ventricle ejection fraction (LVEF) of > 50 % (60.5 % women and 58.3 % men), which did not differ between the sexes (P = 0.26). Regarding laboratory parameters at admission, women had lower haemoglobin level (7.9 ± 1.0 mmol/l vs. 8.8 ± 1.0 mmol/l, P < 0.001), higher platelet count (271 ± 80 *10^9^/μl vs. 239 ± 68 *10^9^/μl, P < 0.001) and lower creatinine level (78 ± 43 μmol/L vs. 95 ± 57 μmol/L, P < 0.001) than men. Furthermore, women had higher total cholesterol level (5.0 ± 1.2 mmol/l vs. 4.7 ± 4.5 mmol/l, P < 0.05) and low-density cholesterol level (3.2 ± 1.1 mmol/l vs. 3.0 ± 1.1 mmol/l, P < 0.001).

**Table 1:** Baseline characteristics for women and men with ACS.

	**Women** **(N = 1463)**	**Men** **(N = 3560)**	**P-value**
**Demographic characteristics**			
Age (years)	69.7 ± 12.4	65.5 ± 11.7	<0.001
Weight (kg)	73.7 ± 14.6	87.3 ± 14.9	<0.001
BMI (kg/m^2^)*	27.1 ± 5.1	27.5 ± 4.0	0.03
Body surface area (m^2^)	1.83 ± 0.2	2.07 ± 0.2	<0.001
**Cardiovascular risk factors (%)**			
Hypertension	903 (61.7 %)	1929 (54.2 %)	<0.001
Diabetes mellitus	293 (20.0 %)	751 (21.1 %)	0.47
Dyslipidaemia	825 (56.5 %)	2014 (56.6 %)	0.56
Current smoking	728 (49.8 %)	2219 (62.3 %)	<0.001
**Comorbidities (%)**			
Atrial fibrillation	143 (9.8 %)	309 (8.7 %)	0.22
Peripheral artery disease	128 (8.7 %)	293 (8.2 %)	0.55
Chronic kidney disease**†**	376 (25.7 %)	659 (18.5 %)	<0.001
Prior stroke or TIA	159 (10.9 %)	315 (8.8 %)	0.03
Prior MI	262 (18.0 %)	847 (23.9 %)	<0.001
Prior PCI	264 (18.1 %)	834 (23.5 %)	<0.001
Prior CABG	111 (7.6 %)	348 (9.8 %)	0.01
Prior bleeding	71 (4.9 %)	184 (5.2 %)	0.9
Active malignancy	35 (2.4 %)	109 (3.1 %)	0.2
Chronic illness×	717 (49.0 %)	1518 (42.6 %)	<0.001
Creatinine (µmol/L)	78.4 ± 43.1	94.9 ± 57.0	<0.001
Haemoglobin (mmol/L)	7.9 ± 1.0	8.8 ± 1.0	<0.001
Leucocyte count (*10^9^/µl)	10.3 ± 3.9	9.7 ± 3.9	0.22
Thrombocyte count (*10^9^/µl)	271 ± 80	239 ± 68	<0.001
Total cholesterol (mmol/L)	5.0 ± 1.2	4.7 ± 4.5	<0.05
LDL-cholesterol (mmol/L)	3.2 ± 1.1	3.0 ± 1.1	<0.001
**Diagnosis at admission (%)**			0.08
- STEMI	555 (37.9 %)	1473 (41.4 %)	
- NSTEMI	773 (52.8 %)	1776 (49.9 %)	
- Unstable angina	135 (9.2 %)	311 (8.7 %)	
Non-obstructive coronary artery disease	197 (13.5 %)	230 (6.5 %)	<0.001
Killip class ≥ II at admission (%)	224 (15.3 %)	399 (11.2 %)	<0.001
Cardiac arrest at admission (%)	36 (2.5 %)	154 (4.3 %)	0.01
LVEF at discharge (%)			0.26
- >50 %	882 (60.5 %)	2069 (58.3 %)	
- 30–50 %	292 (20.0 %)	783 (22.1 %)	
- <30 %	57 (3.9 %)	162 (4.6 %)	
- Unknown	227 (15.6 %)	533 (15.0 %)	

Values are presented as mean ± standard deviation or number of patients (percentage).

* Body mass index, weight and body surface area was missing in 169 cases (3.4%). These cases were excluded from analyses requiring these variables but were retained in all other analyses. Missing values were not imputed.

**†** Chronic kidney disease was defined as a glomerular filtration rate < 60 ml/min/1.73 m^2^.

× Chronic illness was defined as the presence of at least one of the following comorbidities: atrial fibrillation, diabetes, peripheral artery disease, chronic kidney disease or chronic obstructive pulmonary disease (COPD).

**ACS** denotes acute coronary syndrome **BMI** body mass index, **CABG** coronary artery bypass grafting, **LVEF** left ventricular ejection fraction, **MI** myocardial infarction, **NSTEMI** non-ST-segment elevation myocardial infarction, **PCI** percutaneous coronary intervention, **STEMI** ST-segment elevation myocardial infarction and **TIA** transient ischemic attack.

### 3.2 Invasive treatment

Invasive in-hospital treatment for women and men is shown in Table 2. Overall, 92.1 % of women and 95.4 % of men underwent coronary angiography (P < 0.01). Women less frequently underwent interventional treatment, both percutaneous coronary intervention (PCI) (66.3 % vs. 71.0 %, P < 0.01) and coronary artery bypass grafting (CABG) (9.2 % vs. 14.0 %, P < 0.001). After excluding patients with non-obstructive coronary artery disease, the differences in PCI and CABG rates between women and men remained unchanged; PCI (64.3 % vs. 69.5 %, P < 0.01) and CABG (9.2 % vs. 14.0 %, P < 0.001).

**Table 2:** Invasive and pharmacological management of women and men with ACS.

	**Women** **(N = 1463)**	**Men** **(N = 3560)**	**P-value**
Coronary angiography (%)	1348 (92.1 %)	3396 (95.4 %)	<0.001
PCI (%)	942 (66.3 %)	2477 (69.6 %)	0.001
- excluding non-obstructive CAD	941 (64.3 %)	2473 (69.5 %)	<0.001
CABG (%)	134 (9.2 %)	498 (14.0 %)	<0.001
- excluding non-obstructive CAD	134 (9.2 %)	498 (14.0 %)	<0.001
**Pharmacotherapy at discharge (%)**			
Aspirin	1204 (82.3 %)	3059 (85.9 %)	<0.001
P2Y_12_-inhibitor	1316 (90.0 %)	3318 (93.2 %)	<0.001
Oral anticoagulant	244 (16.7 %)	579 (16.3 %)	0.72
Beta-blocker	1055 (72.1 %)	2451 (68.8 %)	0.02
ACE-inhibitor or ARB	1020 (69.7 %)	2628 (73.8 %)	0.003
- ACE-inhibitor	809 (55.3 %)	2214 (62.2 %)	<0.001
- ARB	218 (14.9 %)	437 (12.3 %)	0.01
Cholesterol lowering drug	1297 (88.7 %)	3301 (92.7 %)	<0.001
Proton pump inhibitor	1237 (84.6 %)	2913 (81.8 %)	0.02
All five class I medications	652 (44.6 %)	1718 (48.3 %)	0.02

**ACE** denotes angiotensin-converting enzyme, **ACS** acute coronary syndrome, **ARB** angiotensin receptor blocker, **CABG** coronary artery bypass grafting, **CAD** coronary artery disease, **PCI** percutaneous coronary intervention

### 3.3 Pharmacotherapy at discharge

**Fig. 2:**
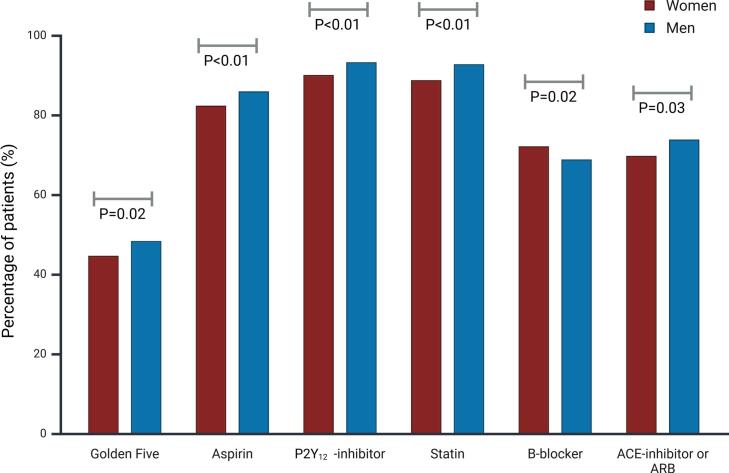
Pharmacotherapy at discharge in women and men with ACS Values are presented as percentage of total patients. **ACE** denotes angiotensin-converting enzyme and **ARB** angiotensin receptor blocker.

Fig. 2 and Table 2 show the pharmacotherapy at discharge for women and men. Overall, 47.2 % of patients received optimal guideline-recommended therapy (i.e., golden five): 44.6 % of women vs. 48.3 % men (P = 0.02). P2Y_12_-inhibitors were the most commonly prescribed drug in both women and men (90.0 % vs. 93.2 %, P < 0.001), followed by cholesterol-lowering drugs (88.7 % vs. 92.7 %, P < 0.001), aspirin (82.3 vs. 85.9 %, P < 0.001), beta-blocker (72.1 % vs. 68.8 %, P = 0.02) and lastly ACE inhibitors or ARB (69.7 % vs. 73.8 %, P = 0.003). Oral anticoagulants were used to a similar degree by women and men (16.3 % vs. 16.7 %, P = 0.72). Each of the guideline-recommended drugs, except for beta blockers, was less frequently prescribed in women. However, women more often received a proton pump inhibitor (84.6 % vs. 81.8 %, P = 0.02).

### 3.4 Antithrombotic regimen at discharge

**Fig. 3:**
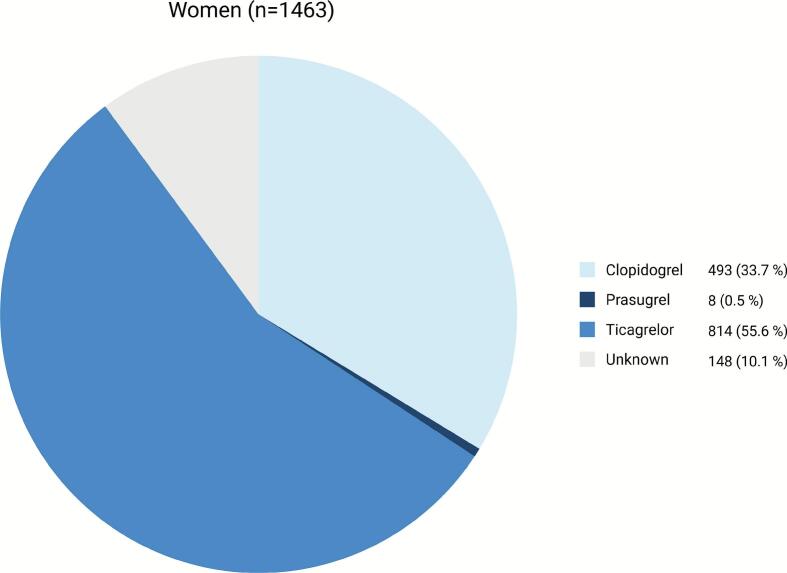
Use of oral P2Y_12_-inhibitors in women with ACS.

**Fig. 4:**
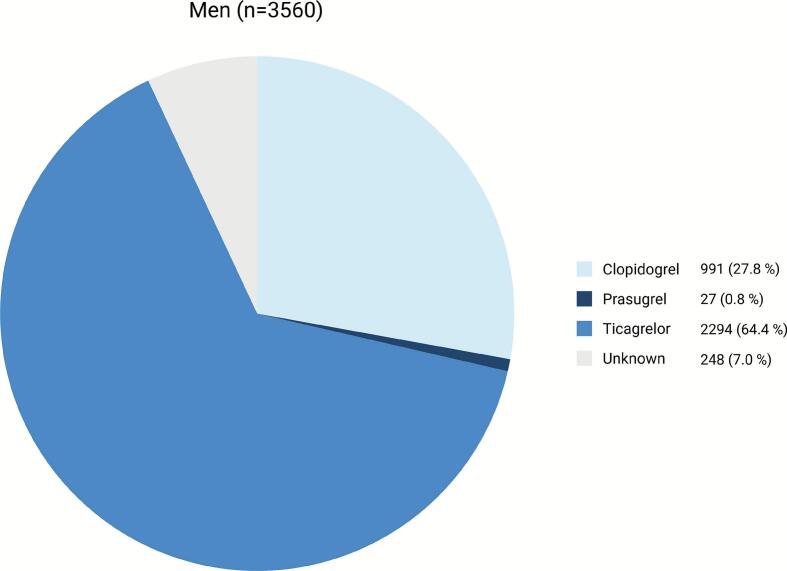
Use of oral P2Y_12_-inhibitors in men with ACS.

**Fig. 5:**
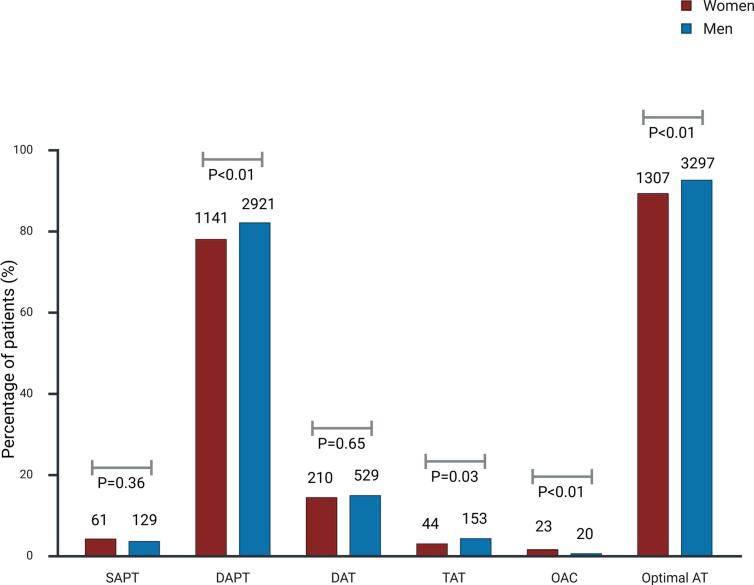
Antithrombotic regimen at discharge in women and men with ACS Values are presented as percentage of total patients. Optimal AT is defined as DAPT, DAT or TAT **AT** denotes antithrombotic therapy, **DAPT** dual antiplatelet therapy, **DAT** dual antithrombotic therapy, **OAC** oral anticoagulant, **SAPT** single antiplatelet therapy and **TAT** triple antithrombotic therapy.

Treatment with different P2Y_12_-inhibitors and combinations of antiplatelet and anticoagulant treatment regimens in women and men, respectively, are shown in Fig. 3, Fig. 4, Fig. 5. Ticagrelor was the most commonly prescribed P2Y_12_-inhibitor in both groups, whereas prasugrel was the least prescribed P2Y_12_-inhibitor. Women were more likely to be prescribed clopidogrel and less likely ticagrelor, compared to men (33.7 % vs. 27.8 % and 55.6 % vs. 64.4 %, respectively, P < 0.01). Aspirin was prescribed in 82.3 % of women and 85.9 % of men (P < 0.001). Also, women less frequently received DAPT (78.0 % vs. 82.1 %, P < 0.001), triple antithrombotic therapy (TAT) (3.0 % vs. 4.3 %, P = 0.03) or optimal antithrombotic therapy (DAPT, DAT or TAT) (89.3 % vs. 92.6 %, P < 0.001), but were more frequently treated with OAC only (1.6 % vs. 0.6 %, P < 0.001).

### 3.5 Clinical outcomes

Clinical outcomes at 12, 24 and 36 months are shown in Table 3, Table 4. The primary endpoint of all-cause death occurred more often in women compared to men (13.7 % vs. 11.0 %, odds ratio [OR] 1.28, 95 % confidence interval [CI]: 1.07–1.54) although sex was not an independent predictor of death after adjustment for age (OR 0.87, 95 % CI: 0.71–1.06) or after full adjustment for significant baseline factors (OR 1.00 95 % CI: (0.81–1.24).

**Table 3:** Clinical outcomes at 12 and 24 months in women and men with ACS.

	**12 months**				**24 months**	
	**Women** **(N = 1463)**	**Men** **(N = 3560)**	**p-value**	**Women** **(N = 1463)**	**Men** **(N = 3560)**	**p-value**
All-cause death	113 (7.7 %)	180 (5.1 %)	0.001	154 (10.5 %)	291 (8.2 %)	0.008
- Cardiovascular	72 (4.9 %)	101 (2.8 %)	0.001	80 (5.5 %)	130 (3.7 %)	0.003
- Non-cardiovascular	27 (2.0 %)	55 (1.6 %)	0.445	41 (2.8 %)	101 (2.8 %)	0.946
Recurrent ACS	78 (5.3 %)	172 (4.8 %)	0.459	124 (8.5 %)	264 (7.4 %)	0.201
Stroke	23 (1.6 %)	35 (1.0 %)	0.076	28 (1.9 %)	60 (1.7 %)	0.575
Major or minor bleeding	306 (20.9 %)	595 (16.7 %)	0.001	347 (23.7 %)	707 (19.9 %)	0.002
Major bleeding	77 (5.3 %)	126 (3.5 %)	0.005	89 (6.1 %)	159 (4.5 %)	0.016
Minor bleeding	243 (16.6 %)	504 (14.2 %)	0.026	282 (19.3 %)	608 (17.1 %)	0.064

Percentages are the cumulative incidence of the clinical endpoints.

**ACS** denotes acute coronary syndrome.

**Table 4:** Clinical outcomes at 36 months in women and men with ACS.

	**Women** **(N = 1463)**	**Men** **(N = 3560)**	**Unadjusted OR**	**p-value**	**Age-adjusted OR**	**p-value**
All-cause death	200 (13.7 %)	392 (11.0 %)	1.28 (1.07–1.54)	0.008	0.87 (0.71–1.06)	0.161
- Cardiovascular	89 (6.1 %)	152 (4.3 %)	1.42 (1.11–1.90)	0.007	1.06 (0.81–1.41)	0.665
- Non-cardiovascular	59 (4.0 %)	150 (4.2 %)	0.96 (0.70–1.30)	0.771	0.66 (0.48–0.91)	0.011
Recurrent ACS	149 (10.2 %)	326 (9.2 %)	1.13 (0.92–1.38)	0.259	1.03 (0.84–1.27)	0.798
Stroke	33 (2.3 %)	88 (2.5 %)	0.91 (0.61–1.37)	0.650	0.77 (0.51–1.17)	0.223
Major or minor bleeding	384 (26.2 %)	793 (22.3 %)	1.24 (1.08–1.43)	0.003	1.12 (0.97–1.29)	0.124
Major bleeding	98 (6.7 %)	182 (5.1 %)	1.33 (1.03–1.71)	0.026	1.07 (0.83–1.39)	0.596
Minor bleeding	319 (21.8 %)	688 (19.3 %)	1.16 (1.00–1.35)	0.046	1.07 (0.92–1.25)	0.363

	Fully adjusted* OR	**p-value**
All-cause death	1.00 (0.81–1.24)	0.983
- Cardiovascular	1.22 (0.91–1.65)	0.180
- Non-cardiovascular	0.73 (0.52–1.02)	0.061
Recurrent ACS	1.16 (0.94–1.44)	0.178
Stroke	0.79 (0.52–1.21)	0.273
Major or minor bleeding	1.15 (0.99–1.33)	0.063
Major bleeding	1.11 (0.85–1.45)	0.465
Minor bleeding	1.11 (0.95–1.30)	0.195

Percentages are the cumulative incidence of the clinical endpoints.

*Fully adjusted (i.e.; for age, current smoking, the presence of at least one concomitant chronic disease, hypertension, chronic kidney disease (CKD), prior MI, prior PCI or prior CABG, MINOCA, cardiac arrest and Killip class ≥ II).

**ACS** denotes acute coronary syndrome and **OR** odds ratio.

Regarding secondary outcomes, cardiovascular death (6.1 % vs. 4.3 %, OR 1.42, 95 % CI: 1.11–1.90) and bleeding (major and/or minor bleeding) (26.2 % vs. 22.3 %, OR 1.24 95 % CI: 1.08–1.43) occurred more often in women, however after adjustment for age and full adjustment for baseline factors, these differences in outcomes were not present anymore. There were no differences in the rate of non-cardiovascular death, recurrent ACS or stroke between women and men (4.0 % vs. 4.2 %, OR 0.96, 95 % CI: 0.70–1.30), (10.2 % vs. 9.2 %, OR 1.13, CI: 0.92–1.38) and (2.3 % vs. 2.5 %, OR 0.91, 95 % CI: 0.61–1.37) respectively.

## 4 Discussion

This study investigated sex differences in the clinical presentation, management and outcomes of patients presenting with ACS. Based on the data of 5023 patients included in a region-wide registry, our findings revealed important disparities between women and men. Specifically, there was a difference in clinical presentation, showing a higher cardiovascular risk profile in women. In-hospital management differed between both groups, as women were treated less frequently according to the ACS guideline recommendations. Moreover, women had a higher risk of all cause death, cardiovascular death and bleeding (major and/or minor), while recurrent ACS and stroke were similar at 36 months compared to men. However, after adjustment for confounders, these differences in clinical outcomes were no longer statistically significant.

### 4.1 Baseline characteristics and risk factors

Consistent to other ACS registries, more men than women were enrolled in this registry [4,12]. A possible explanation for the lower enrolment of women may be their different symptom presentation, which can lead to misdiagnoses, combined with insufficient awareness or a tendency to seek medical care later than men, as demonstrated by previous studies [13,14]. Moreover, women are commonly underrepresented in cardiovascular clinical trials, which may be due to women being less willing to participate in trials [15]. However, since FORCE-ACS is a registry and not an (interventional) trial, the lower enrolment rate cannot be explained by the aforementioned factor. Notably, the women:men ratio in our study was 1:2.4, which is comparable with data from other large registries, such as the Swedish Coronary Angiography and Angioplasty Registry (SCAAR) data on ACS patients (ratio 1:2,7) and the French Registry of Acute ST-elevation or non-ST-elevation Myocardial Infarction (ratio 1:2.8) [16,17].

Similar to most other clinical trials and registries, women in our cohort were slightly older and had more comorbidities, including hypertension and chronic kidney disease than men, contributing to a higher cardiovascular risk profile [17]. The combination of older age and the presence of co-morbidities has been raised as one of the underlying causes of adverse long-term outcomes observed in women after an ACS, compared to men [[18], [19], [20]]. After menopause, women tend to exhibit higher rates of comorbidities compared to men. Menopause is considered as a transition period to a worse cardiovascular risk profile, as circulating oestrogen plays a protective role in maintaining vascular endothelial function [21]. This leads to a higher prevalence of hypertension, diabetes, hypercholesterolemia and change of fat distribution, resulting in a worse cardiovascular risk profile [22]. However understanding the complex relationship between menopause, aging and cardiovascular events remains challenging and is not the topic of this report [23].

### 4.2 In-hospital management and use of medications

Women in our cohort were less likely to undergo interventional diagnostics and treatment, even after exclusion of non-obstructive coronary artery disease. The higher cardiovascular risk profile at admission in our cohort, characterized by a greater prevalence of chronic kidney disease, combined with advanced age and other comorbidities, may explain the lower rate of invasive diagnostics and revascularization observed in women compared to men in our cohort. Furthermore, women enrolled in FORCE-ACS presented with less extensive macrovascular CAD, consistent with previous studies. MINOCA, which occurred in 13.5 % women compared to 6.5 % in men in our cohort, presents a diagnostic and therapeutic challenge due to its heterogeneous causes and lack of standardized treatment [11,24]. However, our data indicate that the higher prevalence of MINOCA in women does not fully explain the observed disparities in revascularisation rates, highlighting the need for further research into sex specific therapeutic strategies to optimize the management of ACS in women.

Although women in our cohort had a lower rate of revascularization, they were also less likely to receive optimal medication for secondary cardiovascular prevention, including platelet inhibitors (e.g., aspirin or P2Y_12_-inhibitor), DAPT, lipid-lowering drug or ACE inhibitor/ARBs compared to men, although these differences were generally small in absolute terms. On the contrary, more women in our cohort were taking beta-blockers, reflecting the higher prevalence of hypertension. In line with our findings, prior studies showed that medical therapy is less frequently given to women on admission and discharge, even though the use of evidence-based classes of cardiovascular medications benefits both women and men [25]. Possible explanation for this might include the older age of female patients, who often present with comorbidities that may lead physicians to be more cautious with certain medications. Additionally, women frequently present with different symptoms or with MINOCA, which may contribute to uncertainty regarding the need for intensive medical therapy.

In our cohort, DAPT was prescribed less frequently in women. While ticagrelor was the most commonly prescribed P2Y_12_-inhibitor, prasugrel, the other potent option, was rarely prescribed in the overall cohort (0.7 %), despite being the first choice P2Y_12_-inhibitor after ACS according to current guidelines due to its superior efficacy to ticagrelor [2]. This low prescription rate of prasugrel is possibly related to the recruitment period: while the new recommendation for prasugrel was published in 2020, our cohort was recruited between 2015 and 2019, prior to the updated guideline. Additional factors, such as contra-indications (e.g., previous stroke) and the need for dose adjustments, might also have limited the use of prasugrel [26].

Numerous studies consistently demonstrate that women with ACS experience higher bleeding rates than men, likely due to sex related differences in body surface area, lower body weight, drug metabolism and pharmacokinetics [19,27]. Women in our cohort had significantly lower body surface area and body weight compared to men. Therefore, antiplatelet therapy must be carefully balanced against the bleeding risk. Accurate dosing and drug selection are crucial to reduce bleeding rates in women. Further research into the pharmacokinetics in women could help optimize the composition and duration of DAPT and minimize complications.

### 4.3 Clinical outcome and mortality

Several studies have examined sex differences in mortality rates and clinical outcomes following ACS. In our cohort, we initially observed a higher mortality rate among women compared to men. Various factors may contribute to this difference, including delayed presentation, lower referral rates for coronary angiography, reduced revascularization rates and lower prescription rate of guideline-recommended medication. Additionally, a higher occurrence of MINOCA may divert clinical attention and has been associated with unfavourable health outcomes [28]. Furthermore, increased bleeding complications and poorer adherence to clinical guidelines could also contribute to higher mortality rates.

However, after adjusting for age and other baseline factors, the differences in mortality and other clinical outcomes were no longer statistically significant in our cohort. This is particularly noteworthy given that, despite having a worse cardiovascular risk profile and receiving less guideline-recommended treatment than men, women did not experience worse outcomes three years after initial admission. Our findings raise the question of whether outcomes in women could be further improved if they were treated optimally according to guideline recommendations or if they received similar treatment as men, highlighting the need for targeted improvements in the management of ACS in women.

Similar to our results, recent analyses from the Get With the Guidelines–Coronary Artery Disease Database and the SWEDEHEART Registry found no sex differences in in-hospital or one-year mortality rates after ACS when adjusted for multiple variables [29,30]. Our findings suggest that the initial disparity in mortality and clinical outcomes among women after ACS might be related to the differences in age, comorbidities and clinical factors, indicating that sex itself is not an independent predictor of poorer outcomes following ACS.

Despite similar long-term mortality rates after adjustment for baseline characteristics, the initial differences highlight the importance of personalized diagnostic and treatment strategies. Further research is essential to better understand the underlying causes of these differences and to develop interventions that optimize outcomes for both women and men with ACS.

## 5 Limitations

There are several limitations to our study. First, the study is limited by the completeness of hospital records. Second, our analysis was based on data collected in the FORCE-ACS registry, so other data which might help to explain the observed findings, such as patient management prior to hospital admission, chest pain characteristics, time intervals from symptom onset to treatment initiation, completeness of revascularization (i.e. number of vessel-disease), dynamic electrocardiographic changes and SYNTAX score were not available for the analysis. Third, since the FORCE-ACS registry is not specifically focused on women, data on female specific risk factors (polycystic ovary syndrome, premature menopause, history of pregnancy-induced hypertension, preeclampsia or gestational diabetes mellitus) were not available as well. Fourth, we did not perform age-stratified analyses. Although age may influence sex-related differences in ACS outcomes, such analyses were not part of the prespecified study design and would have resulted in subgroups too small for reliable estimates. Fifth, the inclusion of heterogeneous ACS subtypes (STEMI, NSTEMI, UA, MINOCA) may have obscured sex-specific differences. However, restricting the analysis to only STEMI or NSTEMI would narrow the scope and remove clinically relevant subgroups—particularly UA and MINOCA, which are known to occur more frequently in women. Therefore, all ACS subtypes were included in the analysis. Sixth, since the pharmacotherapy at follow-up was based on the patient-reported questionnaires and could not be objectively verified, we could not derive any conclusions on the sex specific associations between compliance and 36-month clinical outcomes. Finally, the findings of this study might have been influenced by the Dutch healthcare system, where ACS care is organized through regional networks that ensure rapid triage, timely revascularization and standardized secondary prevention. This may limit generalizability to other healthcare settings.

## 6 Conclusion

Differences between women and men were observed in clinical characteristics, interventional treatment, pharmacotherapy and clinical outcomes among ACS patients in The Netherlands, enrolled in the FORCE-ACS registry. Despite having a higher cardiovascular risk profile and receiving less guideline-recommended care, women had similar adjusted 36-month outcomes as men, which was independent of sex. This study shows that there is room for improvement in the management of ACS, and that better prevention and optimized treatment strategies for women with ACS is urgently needed.

## CRediT authorship contribution statement

**Shabiga Sivanesan:** Writing – original draft, conceptualization. **Aleksandra Gąsecka:** Review & editing, Supervision. **Niels M.R. van der Sangen:** review & editing. **Wout W.A. van den Broek:** review & editing. **Jaouad Azzahhafi:** review & editing. **Dean R.P.P. Chan Pin Yin:** review & editing. **Qiu Ying F. van de Pol:** review & editing. **Ronald J. Walhout:** review & editing. **Melvyn Tjon Joe Gin:** review & editing. **Ron Pisters:** review & editing. **Deborah M. Nicastia:** review & editing. **Gerben J. de Roest:** review & editing. **Georgios J. Vlachojannis:** review & editing. **Rutger J. van Bommel:** review & editing. **Wouter J. Kikkert:** review & editing. **José P.S. Henriques:** Review & editing, Supervision. **Jurriën M. ten Berg:** Review & editing, Supervision. **Yolande Appelman:** Review & editing, Supervision.

## Funding

The FORCE-ACS registry is supported by grants from the Netherlands Organization for Health Research and Development (ZonMw, The Netherlands), the St. Antonius Research Fund and AstraZeneca. The authors are solely responsible for the design and conduct of this study, all study analyses, the drafting and editing of the manuscript and its final contents.

## Declaration of competing interest

The authors declare that they have no known competing financial interests or personal relationships that could have appeared to influence the work reported in this paper.
